# Growth strategies and phenotypic plasticity of improved Chinese fir families across soil types

**DOI:** 10.48130/forres-0025-0022

**Published:** 2025-10-30

**Authors:** Zhen Zhang, Wenyue Wang, Huimin Niu, Haobo Zhao, Jingyong Ji, Guiping He, Zhichun Zhou

**Affiliations:** 1 Research Institute of Subtropical Forestry, Chinese Academy of Forestry, Hangzhou 311400, China; 2 Zhejiang Key Laboratory of Forest Genetics and Breeding, Hangzhou 311400, China; 3 Longquan Forestry Research Institute, Lishui 323000, China

**Keywords:** Multi-generational genetically improved varieties, Fine root functional traits, Variation, Phenotypic plasticity, Genotype-environment interaction

## Abstract

To enhance phenotypic plasticity, it is vital to maximize the genetic growth potential of trees and understand their adaptive responses to environmental conditions. Tree species adapt to dynamic environmental conditions by leveraging the interactions among the environment, genotype, and genotype-by-environment. A total of 25 improved varieties of Chinese fir were transplanted and developed through multi-generational breeding into four types of artificial forest soils. Through a quantitative analysis of genotypic, soil environmental conditions, and genotype-by-environment interaction effects on variations in growth, biomass, and root functional traits, the key drivers of phenotypic plasticity were identified. The results indicate that soil environmental conditions and genotype-by-environment interactions are the primary factors influencing trait variation, explaining 55.89% to 93.94% of the observed variation, while the family effect is relatively minor. Notably, pronounced phenotypic plasticity drives divergent selection in both aboveground and belowground growth strategies. Critical traits influencing root dry weight include root average diameter, total root volume, and root-to-shoot ratio. Although root dry weight does not directly affect plant height, it has a substantial impact on aboveground dry weight. These findings highlight that the changes in the aboveground and belowground growth strategies of Chinese fir during the seedling stage are closely linked to the plasticity of root functional traits. For multi-generational genetically improved varieties, this study examined how the integration of genetic effects, soil environmental conditions, and genotype-by-environment interactions in the selection of aboveground growth and root functional traits influences the mechanisms driving biomass accumulation. The results provide actionable insights for selecting soil-specific families in subtropical plantations.

## Introduction

The declining availability of forest soil resources presents significant challenges in meeting the nutrient demands required for high-quality, high-yield timber production^[1,2]^. In selecting and promoting afforestation species, it is essential to focus on harnessing their growth potential (genotype) and effectively integrating it with environmental factors (e.g., soil resources and climatic conditions), rather than excessively relying on fertilization to enhance productivity^[3−5]^. Consequently, identifying superior genotypes with robust growth potential and high phenotypic plasticity to improve the productivity of artificial forests has become a key research priority in the advancement of artificial forestry^[1]^.

Studies have demonstrated that plants adapt to variations in soil nutrient availability through biomass allocation and adjustments in functional traits, thereby optimizing their utilization of limited resources. The growth potential of trees is influenced by their genotypic responses to variable growth environments, which results in substantial phenotypic variation. When sustained, this phenotypic variation provides a crucial foundation for selecting superior genotypes and evaluating their adaptability^[6]^. Phenotypic evaluation trials involving multiple genotypes across diverse environments are essential for identifying superior genetic materials in plant breeding^[7−9]^. However, plant growth exhibits diverse responses to limited soil nutrients, with root systems serving as vital resource-acquisition organs that determine the quantity of water and nutrients absorbed for photosynthesis and growth^[4,10−13]^. The variation in root traits is extensive, with interspecific variation in absorption root diameter exceeding 100-fold and intraspecific variation exceeding tenfold^[14]^. Therefore, understanding the extent of root trait variation is vital for comprehending root systems and plant adaptation to environmental heterogeneity. Meanwhile, root functional traits should remain a long-term focus for breeders^[3,15]^.

Within tree species, significant variations in root morphology and architecture exist among different genotypes. These differences are primarily reflected in the plasticity of traits such as root branching intensity, fine root length, root surface area, and root volume^[16−18]^. Plant roots have evolved various strategies for nutrient acquisition, particularly for phosphorus, including elongation and thinning to increase surface area and the development of complex branching structures to explore larger soil volumes^[19−22]^. In adverse environments, plants may adapt by increasing root-to-shoot ratios or total root length, reducing root diameter and tissue density to lower construction costs, and expanding absorption areas to meet environmental challenges^[23−25]^. Under resource stress, any trait response can alter a plant's perception of resource limitations, reflecting substantial variability in root plasticity^[9,26,27]^. Furthermore, the high variation in specific root traits among genotypes is critical for developing nutrient foraging strategies. The joint variation of root morphological and functional traits provides valuable insights into the underground nutrient acquisition strategies of plants^[28]^. For instance, plants with finer roots often rely more on morphological adaptations, such as increased specific root length, to expand soil exploration and enhance nutrient uptake, exemplifying a resource-acquisition strategy.

In practice, certain families that perform well in specific environments may exhibit poor phenotypes in other settings, while families with suboptimal performance under one set of conditions may excel in different contexts. This phenomenon is primarily driven by genotype-environment interaction effects (G × E)^[29,30]^. When analyzing the relationship between genotype and environment, the focus is typically on how an organism's genetic makeup interacts with external environmental factors to influence phenotype^[23,27]^. Research on G × E effects has been conducted for numerous commercially important tree species worldwide, including *Pinus elliottii*, *Pinus taeda*, *Picea abies*, *Pinus radiata*, and *Larix kaempferi*^[30−33]^. Most studies indicate that G × E effects are widespread in forestry, highlighting the importance of underscoring the patterns and scales of these effects for accurate assessments of genetic gain in tree traits. Leveraging genotype effects, G × E effects, and soil environmental conditions to identify superior genotypes has proven effective in enhancing yields^[34]^. These studies predominantly focus on evaluating growth rates and wood properties using genetic population materials. However, there is limited research addressing the genetic effects, soil effects, and G × E effects on both aboveground growth potential and root development in trees when faced with varied soil environments. This gap in evaluation carries significant implications for the selection of superior families and the quantitative assessment of soil resource utilization in forestry management.

The promotion of planting forest tree species that have undergone multiple rounds of selection has demonstrated significant growth potential. However, the understanding of how genotype-environment interactions (G × E) affect both the aboveground and root systems, particularly in relation to soil, remains limited. This study aims to investigate how multi-generational selected tree varieties sustain their growth potential under varying soil environmental conditions, focusing on Chinese fir (*Cunninghamia lanceolata* [Lamb.]), which is the most extensively planted tree species in China. It is widely distributed across southern provinces and primarily used for timber and construction materials^[35]^. Twenty-five varieties were transplanted and selected through three breeding generations into four distinct artificial forest soils, and their growth and root functional traits were measured. The objectives of this research are: (1) to elucidate the phenotypic basis underlying the aboveground and belowground growth strategies of different Chinese fir varieties; (2) to assess the relative contributions of genotype, environment, and their interactions to phenotypic variation in growth, biomass, and root functional traits; and (3) to quantify the primary factors driving phenotypic plasticity. By examining the integrated phenotypic responses of Chinese fir varieties to changes in soil environment, this research aims to reveal how these varieties maintain high productivity while adapting to variable soil conditions. The findings will provide valuable insights into the adaptation strategies of Chinese fir and offer a reference framework for its promotion and cultivation across diverse soil environments.

## Materials and methods

### Experimental materials

The materials included 25 families of Chinese fir, which were derived from a zygotic family (open-pollinated) in the third-generation seed orchard, corresponding to parents with diverse genetic backgrounds. These families were selected through rotational breeding for traits such as growth rate, wood density, and stress tolerance (Supplementary Table S1). Each genotype reflects unique combinations of alleles accumulated over three generations of selection, thereby ensuring a broad genetic spectrum for evaluating plasticity.

**Figure 1:**
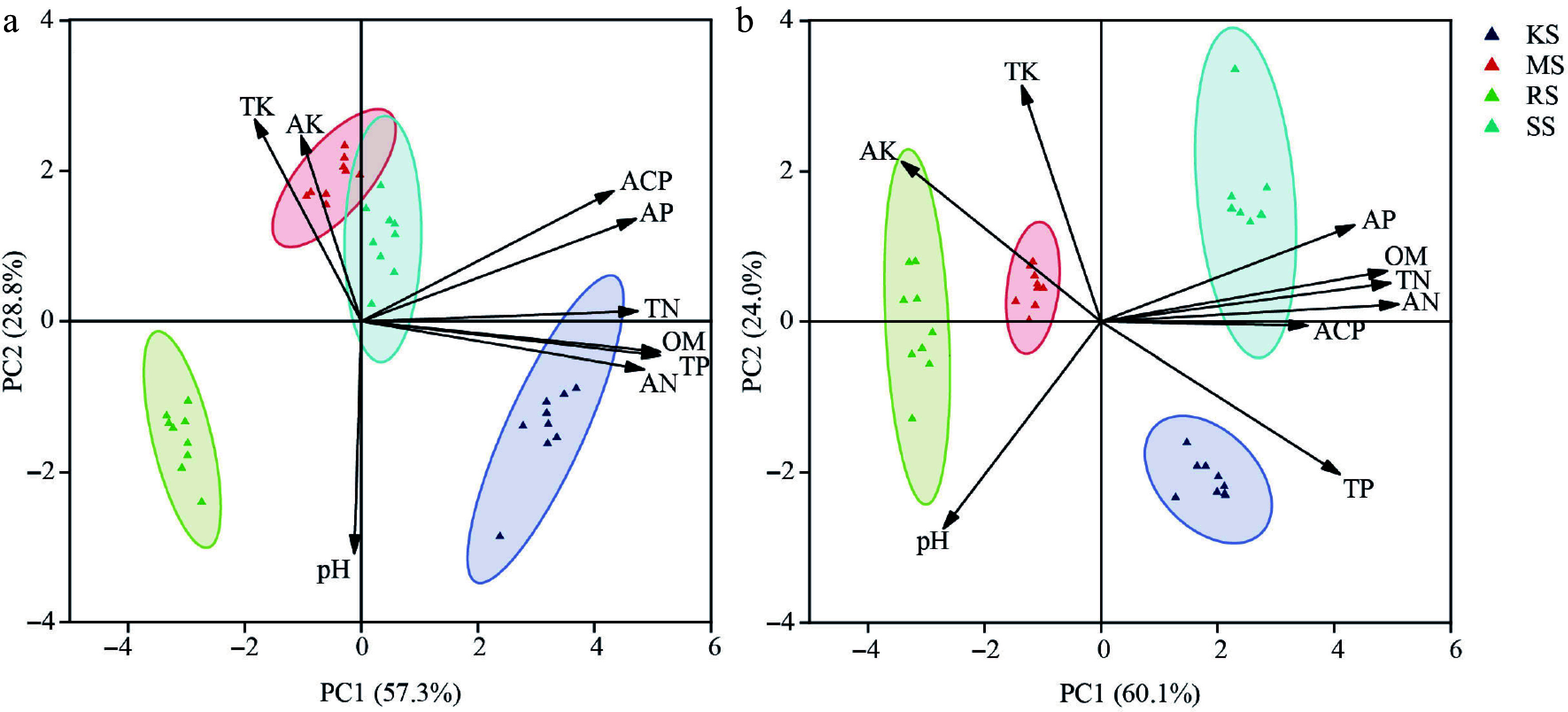
Diversity differences among four soil conditions. (a) Pre-treatment. (b) Post-treatment. The different colored circles in the diagram represent different soil types; TN, total nitrogen; TP, total phosphorus; TK, total potassium; AN, acute nitrogen; AP, available phosphorus; AK, acute potassium; OM, organic matter; ACP, acid phosphatase; pH, acidity and alkalinity.

The controlled potting experiment utilized non-woven bags with a height of 30 cm and a diameter of 20 cm. The soils used for potting were sourced from a pure Chinese fir plantation (SS), a pure *Pinus massoniana* plantation (MS), a pure *Schima superba* plantation (KS), and a common red soil (RS) from an unplanted stand. The selection of these soils was informed by the silvicultural needs of fir trees in production, representing the typical cultivation environment for Chinese fir in subtropical plantations^[36]^. Based on standard pit dimensions for Chinese fir afforestation (60 cm length × 60 cm width × 40 cm depth), soil samples were collected from the 20–40 cm depth layer in plantation forests to accurately reflect root zone conditions. After removing debris (e.g., plant litter, detritus, and gravel), the soils were sieved through a 5 mm mesh. The physicochemical properties and enzymatic activities of the four soil types were quantified, and these exhibited significant variability (Fig. 1). Subsequently, the soils underwent a five-day solarization process by spreading them in thin layers under intense sunlight, with regular turning to ensure uniform exposure.

### Experimental design

Following seed collection in November 2021, seedlings were uniformly germinated and nurtured in seedbeds under standard nursery protocols for one year before transplantation in October 2022. At this stage, the seedlings reached an average height of 21.15 ± 1.22 cm and basal diameters of 2.53 ± 0.19 mm. Each seedling was individually transplanted into non-woven fabric containers, maintaining a 10 cm inter-pot spacing within a climate-controlled greenhouse that employed a split-plot design. This design included 12 blocks representing four soil treatments, with each treatment randomly assigned to three blocks as biological replicates. Within each block, 25 tested families were randomized, with ten seedlings per family arranged linearly, culminating in a total of 3,000 seedlings. Post-transplant watering was thoroughly administered under consistent greenhouse conditions, which maintained daytime temperatures between 21–25 °C, nighttime temperatures between 15–18 °C, a daily photoperiod of 12–14 h, and stabilized humidity levels throughout the growth period.

### Growth survey, sample harvesting, and indicator measurement

#### Growth index survey

Seedling plant height (H) was measured monthly, beginning on April 1, 2023, with an accuracy of 0.01 cm. The final measurement of seedling height was conducted on November 1, 2023. The growth curve of seedling height was derived by fitting the relationship between plant height and planting days using the logistic equation was $ y=\dfrac{k}{1+a{e}^{-bt}} $, where *t* represents the number of growth days, *y* is the growth of seedling height, *k* is the theoretical upper limit of growth, and a and b are coefficients to be determined. The start and end times of the rapid growth period were calculated after deriving the fitted equation with the following formulas: start time of the rapid growth period, *t*_1_ = (ln*a* − 1.317)/*b*, and end of the rapid growth period, *t*_2_ = (ln*a* + 1.317)/b^[37]^.

#### Determination of functional traits of root system

At the conclusion of the survey, whole plant sampling was conducted on all experimental seedlings using the destructive sampling method. The root systems were carefully collected to ensure their integrity before being transported to the laboratory. The underground portion was severed at the root base, after which the root systems were rinsed with deionized water and allowed to dry. The length, surface area, and root volume data of the root systems at each diameter level were analyzed using the image analysis software WinRHIZO Pro STD1600+ (Regent Instruments, Canada). The diameter classifications were as follows: 1^st^ diameter level (D1, 0 to 0.5 mm), 2^nd^ diameter level (D2, 0.5 to 1.0 mm), 3^rd^ diameter level (D3, 1.0 to 1.5 mm), 4^th^ diameter level (D4, 1.5 to 2.0 mm), and 5^th^ diameter level (D5, > 2.0 mm). Parameters such as total root length (RL, cm), total root surface area (SA, cm^2^), total root volume (RV, cm^3^), mean root diameter (RAD, mm), number of bifurcations (RBN), and root conformation grading were obtained. These parameters were subsequently converted to yield specific root length (SRL, cm/g), specific root area (SRA, cm^2^/g), and branching strength (RBS) using the following equations: SRL (cm/g) = RL/RDW, SRA (cm^2^/g) = SA/RDW, and RBS = RBN/RL. Additionally, root systems with a diameter class of ≤ 2.0 mm were classified as fine roots, and the fine root length (TRL, cm), fine root surface area (TRA, cm^2^), and fine root volume (TRV, cm^3^) were calculated.

The aboveground portions were categorized into leaves, stems, and branches, which were then placed in a constant temperature oven at 105 °C for approximately 30 min, followed by drying at 80 °C until a constant weight was achieved. This process allowed for the determination of leaf dry biomass (LDW, g), stem dry biomass (SDW, g), branch dry biomass (BDW, g), above-ground dry biomass (ADW, g), and root dry biomass (RDW, g), from which the root-crown ratio (R/S) was calculated as RDW/ADW.

### Data statistics and analysis

The first step in statistical analysis was to perform the ANOVA according to the model:

$ {y}_{ijk}=\mu +{S}_{ i}{+{R}_{ j\left(i\right)}+F}_{ k}+{F S}_{ ik}+{e}_{ijk} $

where, *y*_*ijk*_ is the individual tree phenotype, *µ* is the overall mean, *S*_*i*_ is the effect of soil environments *i*, *R*_*j(i)*_ is the effect of replication *j* within the site *i*, *F*_*k*_ is the effect of family *k*, *FS*_*ik*_ is the family × soil environments interaction effect, and *e*_*ijk*_ is the error. The same model was fitted with all random effects for estimation of variance components, and then the environment variation coefficient (*CV*_*e*_), genetic variation coefficient (*CV*_*g*_), and genetic correlation coefficient (*CC*_*g*_). Then, family heritability ($ {h}_{f}^{2} $) was calculated according to the formula given by Yuan et al.^[33]^. The least significant difference multiple comparison method was used to analyze the difference levels (with a significance level of *α* = 0.05).

Following the ANOVA, the analysis of GGE (Genotype main effects plus Genotype-by-Environment interaction) biplots was conducted using the R software package GGE-Biplot GUI. Initially, the random effects solution was computed, specifically the BLUPs of the 25 genotypes and their interactions with all environments, based on the previously described model. Subsequently, a double-entry matrix was constructed with genotypes represented as rows and environments as columns^[38]^. Most available software utilizes singular value decomposition to derive the principal components (PCs)^[39,40]^, herein, the model adopted follows the expression $ \dfrac{{Y}_{ij}-\mu -{\beta }_{j}}{{d}_{j}}={\lambda }_{1}{g}_{i1}{e}_{1j}+{\lambda }_{2}{g}_{i2}{e}_{2j}+{\varepsilon }_{ij} $, where *Y_ij_* represents the genetic value of genotype i combined with trait *i* and *j*, *μ* is the average value of all combinations of trait *j*, *β_j_* is the main effect of trait *j*, *g_i1_* and *g_i2_* are the eigenvectors of genotype *i* on principal component PC1 and PC2 respectively, *e_1j_* and *e_2j_* are the eigenvectors of trait j on principal component PC1 and PC2 respectively, *d_j_* is the phenotypic standard deviation, and *ε_ij_* is the model residual resulting from the combination of genotype *i* and trait *j*.

To investigate the effects of genotype and environment on seedling growth, an initial structural equation model (SEM) was conceptualized based on theoretical understanding and prior regression analyses. Genotype and environment were specified as exogenous variables. While plant growth traits and biomass allocation were treated as endogenous mediating variables. Aboveground dry weight (ADW) and root dry weight (RDW) were designated as the final endogenous response variables. The model was implemented and refined using the piecewise SEM package in R software. Non-significant paths (*p* > 0.05) identified through path coefficient tests were sequentially removed. Concurrently, variables with high residual covariance, indicating potential missing paths or shared influences, were evaluated and removed if they did not significantly contribute to the model's explanatory power or theoretical coherence. The final model was evaluated for adequate fit based on the following criteria: a non-significant Fisher's C test (*p* > 0.05), a low Akaike Information Criterion (AIC) value, Root Mean Square Error of Approximation (RMSEA) < 0.05, and Goodness-of-Fit Index (GFI) > 0.95.

## Results

### Variation analysis

**Figure 2:**
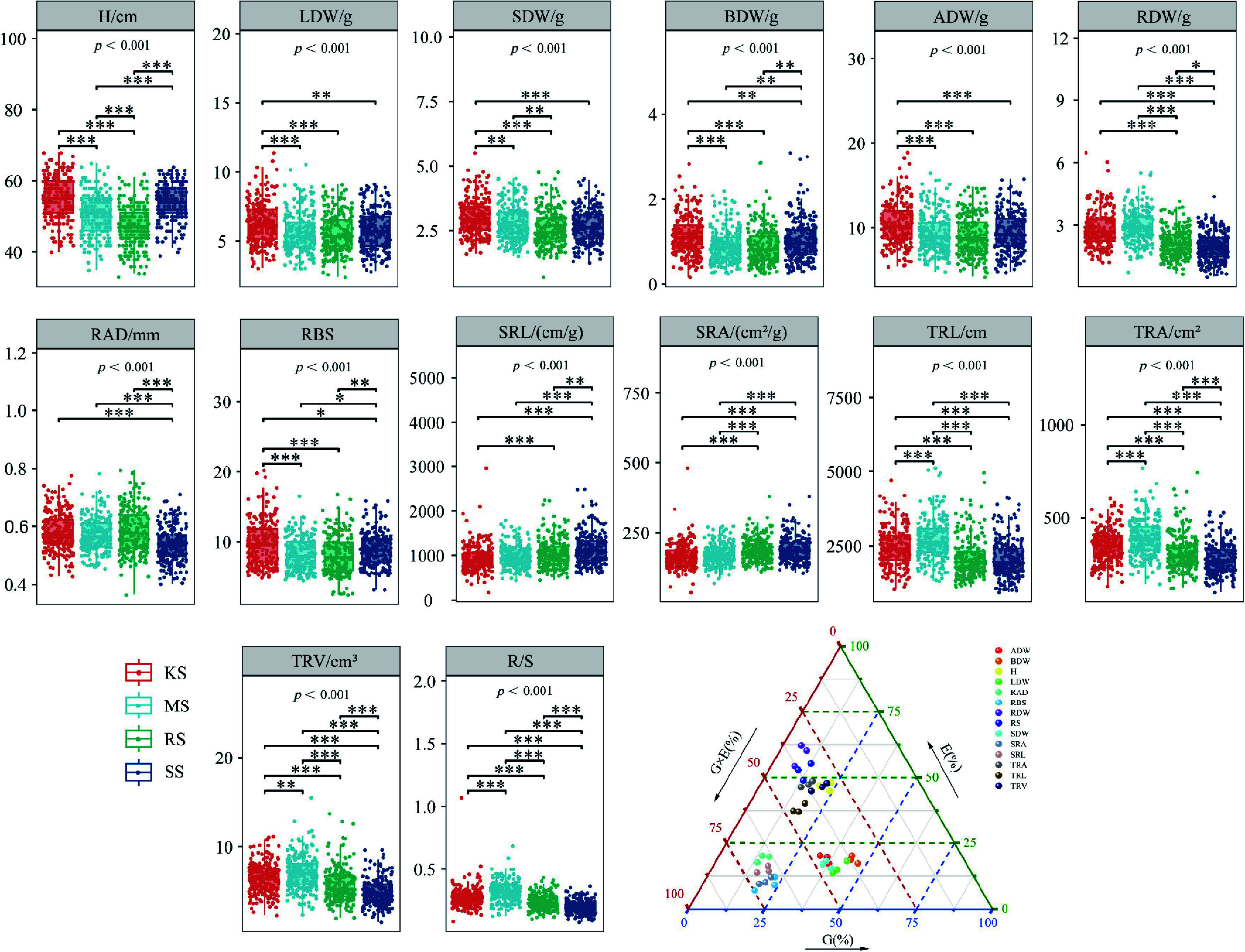
Expression values of traits and contribution rates of variation sources under various soil conditions. Box plots indicate variability in plant height, biomass, and root metrics, and dots indicate individual data, *p* values represent overall differences between groups, the horizontal lines represent comparisons between the two groups at either end of the scale, * indicates the level of significance of differences obtained by two-by-two comparisons; H, Plant Height; ADW, Aboveground dry weight; LDW, Leaf dry weight; BDW, Branch dry weight; SDW, Stem dry weight; RDW, Root dry weight; SRL, Specific root length; SRA, Specific root area; RAD, Root average diameter; RBS, Root branching strength; TRL, Fine root length; TRA, Fine root surface area; TRV, Fine root volume; R/S, Root to shoot ratio; The ternary plot represents the share of genetic variance components, environmental variance components, and variance components of genotype-environment interactions for each of these metrics, with dots of the same color indicating replicate values for that metric. * *p* < 0.05; ** *p* < 0.01; *** *p* < 0.001.

The *CV*_*g*_ for plant height and biomass of each organ ranged from 3.58% to 13.92%, while the *CV*_*e*_ ranged from 9.10% to 40.78%. The genetic variation coefficient for root phenotypic traits varied between 0.91% and 7.20%, with the environmental variation coefficient ranging from 15.50% to 25.87%. These findings suggest that the observed variations in phenotypic traits are influenced by soil environmental conditions, genotype effects, and G × E interactions (Table 1; Fig. 2).

**Table 1:** ANOVA tests for growth and root traits of Chinese fir seedlings.

Traits	Mean value	*F* value				Coefficient of variation (%)			*h*_*f*_^2^
		*G*	*E*	*G × E*		*CV*_*g*_	*CV*_*e*_		
H/cm	51.86	9.75**	144.99**	4.18**		3.58	9.10		0.57
ADW/g	9.57	4.79**	17.45**	1.91**		6.56	23.25		0.60
LDW/g	5.81	4.92**	13.16**	1.84**		6.91	23.61		0.63
BDW/g	0.99	5.90**	19.94**	1.66**		13.92	40.78		0.72
SDW/g	2.77	5.76**	21.77**	2.54**		6.66	22.11		0.56
RDW/g	2.47	1.27**	103.99**	2.22**		4.69	28.80		0.30
SRL/(cm/g)	1,030.05	4.38**	30.54**	6.54**		5.85	23.90		0.25
SRA/(cm^2^/g)	179.50	5.84**	23.90**	7.54**		3.87	17.81		0.18
RAD/mm	2.53	3.69**	37.35**	6.06**		2.73	16.15		0.11
RBS	8.75	21.68**	66.12**	29.46**		7.20	15.50		0.21
TRL/cm	2,338.59	3.35**	63.00**	3.30**		0.91	24.69		0.10
TRA/cm^2^	331.90	3.36**	80.14**	2.60**		3.60	24.86		0.23
TRV/cm^3^	6.04	4.07**	80.57**	2.77**		4.91	25.87		0.32
R/S	0.26	3.75**	163.74**	4.82**		3.88	21.07		0.23
H, Plant Height; ADW, Aboveground dry weight; LDW, Leaf dry weight; BDW, Branch dry weight; SDW, Stem dry weight; RDW, Root dry weight; SRL, Specific root length; SRA, Specific root area; RAD, Root average diameter; RBS, Root branching strength; TRL, Fine root length; TRA, Fine root surface area; TRV, Fine root volume; R/S, Root to shoot ratio. *G*, Genotype interactions; *E*, Environment interactions; *G × E*, Genotype and environment interactions; *CV*_*g*_, Genetic variation coefficient; *CV*_*e*_, Environment variation coefficient; *h*_*f*_^*2*^, Family heritability. ** indicates a significant difference at *p* < 0.01.									

The H, RDW, TRA, TRV, and R/S are significantly influenced by soil environmental conditions, with a variance contribution rate ranging from 44.83% to 62.10%. Conversely, LDW is predominantly affected by genotype (44.11%), while other traits are largely influenced by the G × E interaction, which accounts for 45.23% to 74.69% of the variance, particularly in SRL and SRA. The RAD and RBS are also primarily affected by the G × E interaction, which contributes over 67.90% to their variation, indicating that genotype performance varies significantly across different soil types. Both the G × E interaction and soil environmental conditions are critical factors influencing the growth and root functional traits of fir during the seedling stage, contributing cumulatively 55.89% to 93.94% of the observed variation (Fig. 2).

### Plasticity analysis

**Figure 3:**
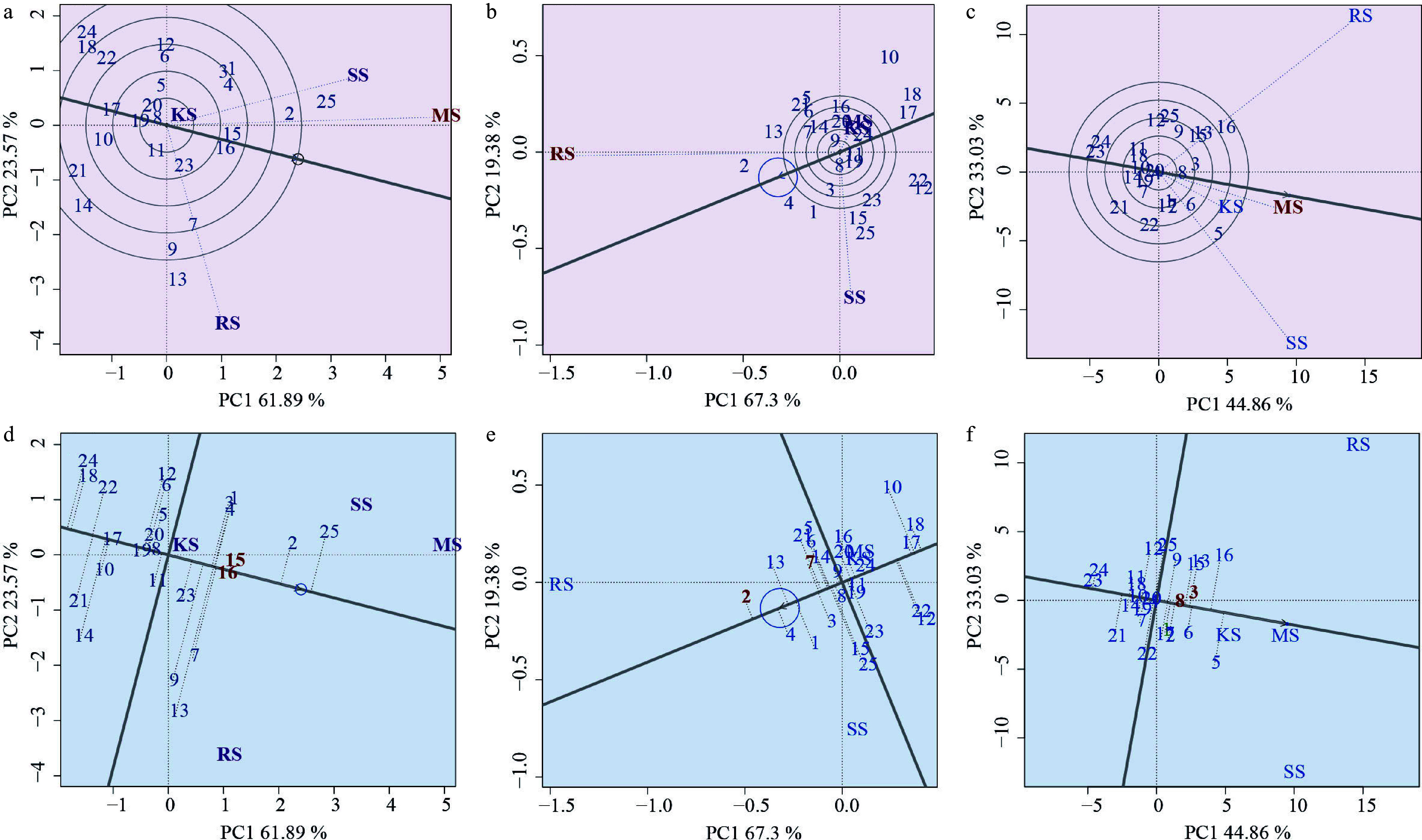
Plot of rank order effects of different family among soil environments. (a), (b), and (c) Representative differentiability and representativeness based on aboveground biomass, root biomass and plant height traits; Where the line with the arrow is the Average Environment Axis (AEA), through the connection between the average environment (the circle in front of the arrow) and the center point. The angle between the line segment of a test point and the Average Environment Axis is a measure of its representativeness of the target environment; the smaller the angle, the more representative it is, and the longer the line segment, the stronger the discriminatory power. If the angle between a test point and the mean environment axis is obtuse, it is not suitable as a test point. The direction of the arrow on the mean environmental axis is an evaluation of both the discriminatory power and representativeness of the test site. (d), (e), and (f) representative stable lineage ranking map based on plant height, aboveground biomass and root biomass traits. The high-yield and stable-yield performance plot requires an Environmental Mean Axis (EMA) represented by a straight arrowed line, as well as Mean Environmental Values (MEV) indicated by circles on the EMA. Additionally, a perpendicular line passing through the origin and orthogonal to the EMA is included. Each variety point is projected onto the EMA using a perpendicular line. The direction of the EMA indicates the approximate average yield trend of the varieties across all environments. The perpendicular line passing through the origin and orthogonal to the EMA represents the tendency of genotype-by-environment interactions (G × E). The longer the perpendicular line between a variety point and the EMA, the less stable the variety is. The numbers in the plot represent different varieties.

This study focused on three key metrics: ADW, RDW, and H. The GGE-BLUP analysis results supported the rank effect of different families among soil environments. PC1 and PC2 collectively account for 85.46% of the G + GE effect on ADW, 86.68% on RDW, and 77.89% on H. As shown in Fig. 3, the variables represent the adaptability of plant height, aboveground biomass, and root biomass of each family. The results show that the more diverse the soil environment, the greater the change in the G × E effect. Given that optimal genotypes exhibit location-specific performance rather than universal adaptability across all four soil environments (reflecting differential phenotypic plasticity), superior families were selected at a 10% selection intensity based on stable and outstanding growth performance under heterogeneous conditions. Specifically, Families 15 and 16 excelled in ADW, Families two and seven dominated RDW, while Families three and eight demonstrated maximal plant height. These selections resulted in mean genetic gains of 4.05% for ADW, 6.32% for RDW, and 4.07% for plant height (Fig. 3).

### Responses of growth and biomass to the environment

**Figure 4:**
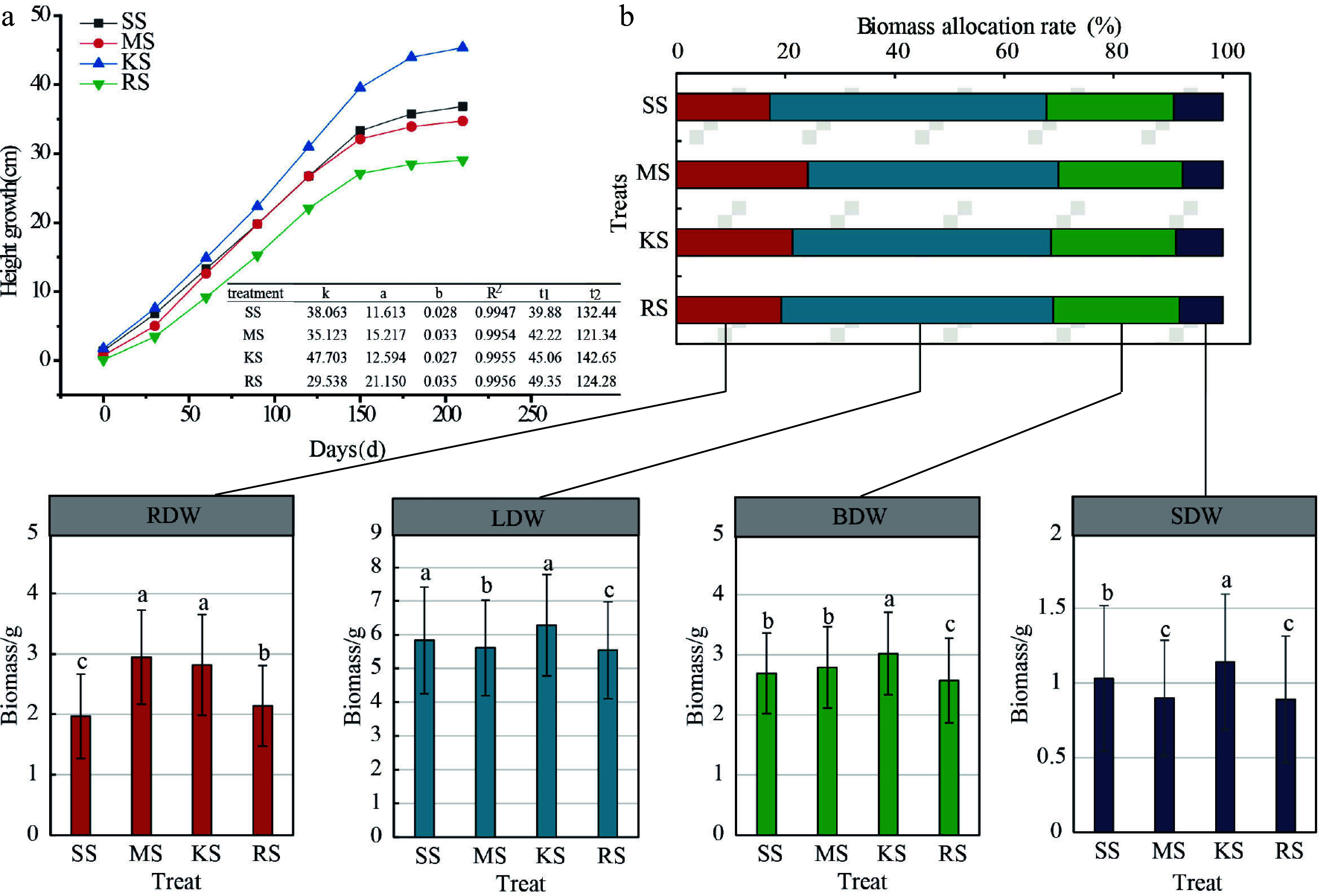
Plant height growth curve and biomass allocation of cedar under different soil treatments. (a) Growth rhythm map, where k is the theoretical limit value of the upper limit value of the growth, and a and b are the coefficients to be determined, *R*^*2*^: coefficient of determination represents the goodness of fit of the model, reflecting the extent to which the independent variable explains the variation in the dependent variable.t_1_: the start time of the rapid growth period; t_2_: end of rapid growth period. (b) Biomass distribution graph. The horizontally arranged bar chart represents the biomass allocation proportions among root, stem, trunk, and leaf organs, while the vertically arranged bar chart indicates the variation in biomass measurements of each organ across treatments; Different lowercase letters denote significant differences between treatments. ADW: Aboveground dry weight; LDW: Leaf dry weight; BDW: Branch dry weight; SDW: Stem dry weight; RDW: Root dry weight.

The growth rhythm of plant height indicates that various plantation soil conditions significantly affect the duration of the rapid growth stage in seedlings, thereby influencing height growth during this phase. The onset of rapid height growth consistently occurs around May 20^th^. In KS soil, the increment in plant height is the highest, and the duration of the rapid growth period is the longest at 98 d. SS soil follows with a slightly lower growth increment; however, its rapid growth period begins the earliest, lasting 93 d. In contrast, the growth increments in MS and RS soils are relatively smaller, with their rapid growth durations being shorter at 79 and 75 d, respectively. Notably, the rapid growth period in KS soil exceeds that of RS soil by 23 d, with an average growth amount 17.43% higher (Fig. 4a). Regarding the ADW, LDW, BDW, and SDW, the ranking among different plantation soils is as follows: KS > SS > MS > RS (Fig. 2). The ADW, LDW, BDW, and SDW in KS soil are higher than those in RS soil by 15.87%, 13.36%, 20.09%, and 17.51%, respectively. In terms of RDW, the order is: MS > KS > RS > SS. with MS soil exhibiting the highest RDW, which is 49.75% greater than that of SS soil (Fig. 4b).

### Responses of above-ground and subsurface growth strategies

The Chinese firs in KS soil belong to a class characterized by H and ADW accumulation, with the RAD significantly increased by 1.75% to 7.41% compared to other soil types. Furthermore, the RBS exhibited the strongest performance, exceeding other soils by 10.29% to 20.52%. In contrast, SS soils, which had the lowest root biomass, demonstrated higher SRL and SRA, along with finer RAD, TRL, TRA, TRV, and R/S compared to other soil types. The RDW in MS soil was the highest, and the indices of TRL, TRA, TRV, and R/S were also at their peak (Fig. 2).

**Figure 5:**
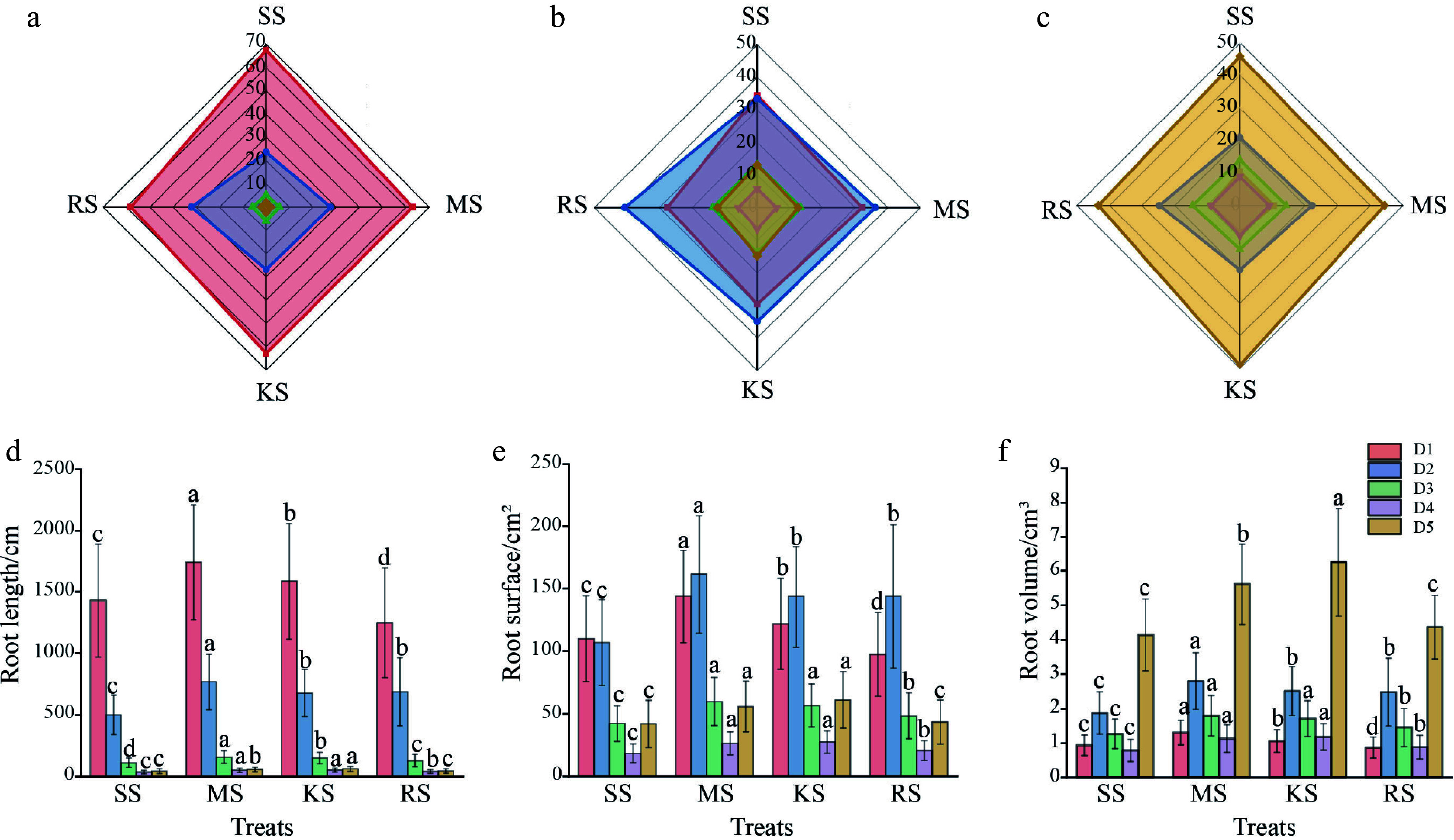
Distribution ratio and growth of D1–D5 radial root classes. (a) Proportion of root length grading (%). (b) Proportion of root surface area grading (%). (c) Proportion of root volume grading (%). (d) Root length. (e) Root surface. (f) Root volume. D1–D5 represent diameter levels, D1: 0 to 0.5 mm, D2: 0.5 to 1.0 mm, D3: 1.0 to 1.5 mm, D4: 1.5 to 2.0 mm, D5: > 2.0 mm.

The proportion of fine root length to total root length ranges from 97.83% to 98.03%, while the proportion of fine root surface area to total root surface area varies from 85.14% to 87.76%. Additionally, the proportion of fine root volume to total root volume ranges from 50.89% to 56.66%. Fine root phenotypic traits appear to be significant factors influencing the differences in underground biomass among various soils (Fig. 5). Notably, the length of fine roots in diameter class D1 accounts for 58.13% to 67.49% of the total root length, with the highest proportion observed in the SS treatment (Fig. 5a), which is 7.43%, 7.37%, and 16.10% greater than that in MS, KS and RS soils, respectively. The fine root length proportions for diameter classes D2 and D3 range from 23.62% to 32.06% and 5.31% to 6.02%, respectively, both exhibiting a higher proportion in RS soil, whereas the proportion of fine root length in diameter classes D4 and D5 are greatest in KS soils (Fig. 5a). A similar trend is observed in the proportion of root surface area across each diameter class in different soils (Fig. 5b, c). This suggests that seedlings in relatively nutrient-poor soils depend more on the increased proportions of length and surface area of fine roots in diameter classes D1, D2, and D3 to enhance their ability to absorb soil nutrients.

**Figure 6:**
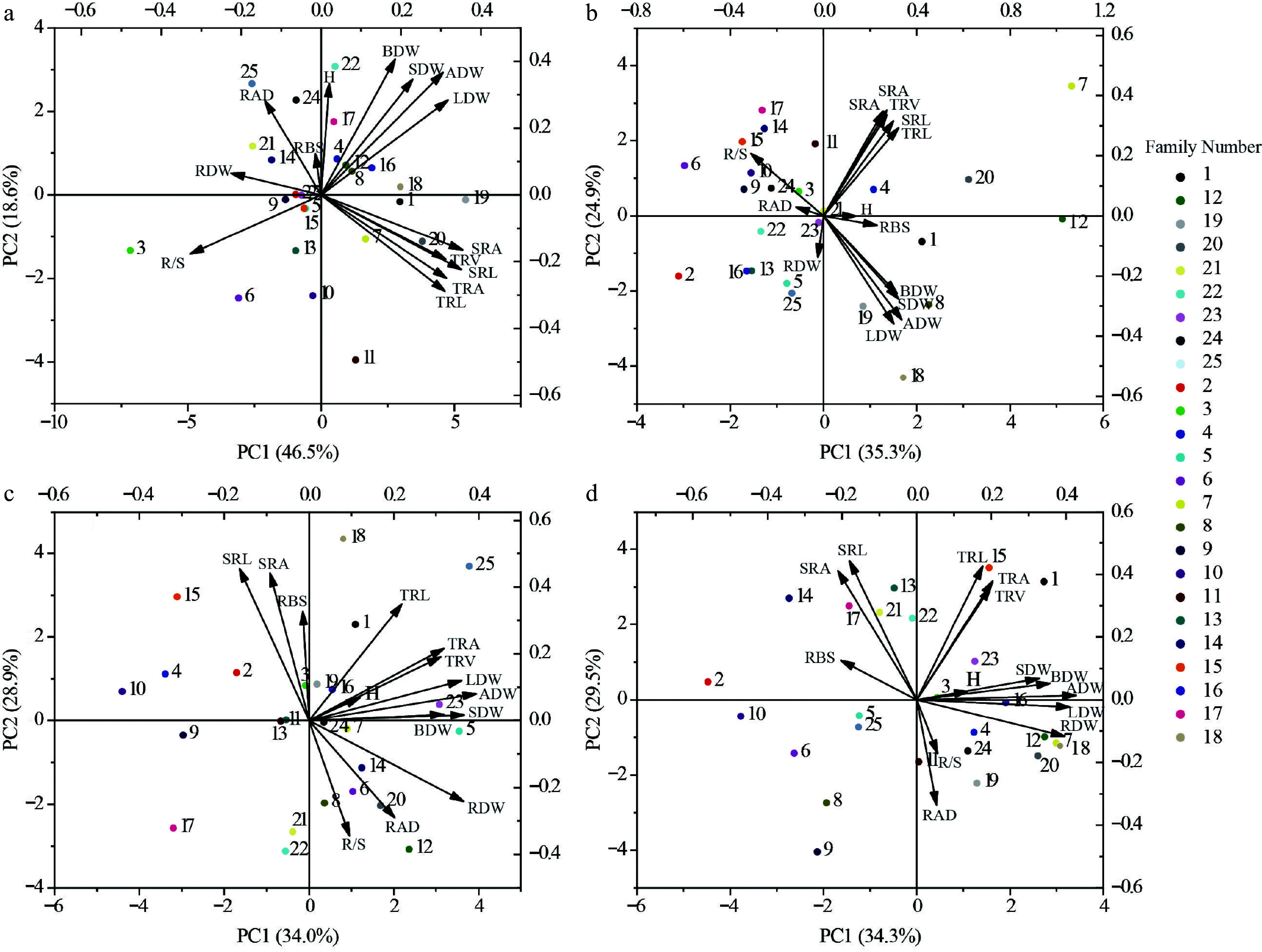
Selection of different genotypes for growth in each of the four soils. (a) SS Soil conditions. (b) MS Soil conditions. (c) KS Soil conditions. (d) RS Soil conditions. The numbered points in the diagram represent different families of Chinese fir. H, Plant height; ADW, Aboveground dry weight; LDW, Leaf dry weight; BDW, Branch dry weight; SDW, Stem dry weight; RDW, Root dry weight; SRL, Specific root length; SRA, Specific root area; RAD, Root average diameter; RBS, Root branching strength; TRL, Fine root length; TRA, Fine root surface area; TRV, Fine root volume; R/S, Root to shoot ratio. Different colored circles represent different family.

The R/S ratios for each soil type range from 0.2065 to 0.3237, with the R/S values in SS and RS soils being relatively lower. Specifically, the R/S ratio for SS soil is 67.62% and 33.61% lower than those for MS and KS soils, respectively. This indicates that under nutrient-limited soil conditions, the accumulation and proportion of RDW are relatively low, which correlates with resource utilization in relation to the biomass allocation of aboveground leaf, branch, and stem parts (Fig. 4b). In terms of organ biomass proportion, LDW exhibits the highest proportion, ranging from 45.80% to 50.61%, followed by branch, root, and stem organs. SS soils display the highest biomass proportion in leaf, branch, and stem organs and the lowest in root biomass. Compared to KS soil, which has the highest total biomass accumulation, the root biomass proportion in SS soils is 4.17% lower. These results suggest that different plantation soils influence the alteration of the aboveground and belowground growth patterns of Chinese fir seedlings. The combination of root traits employs diverse strategies to enhance adaptability, and a trade-off mechanism exists between growth and nutrient acquisition (Figs 4b & 6).

### Analysis of character covariance and influencing factors

**Figure 7:**
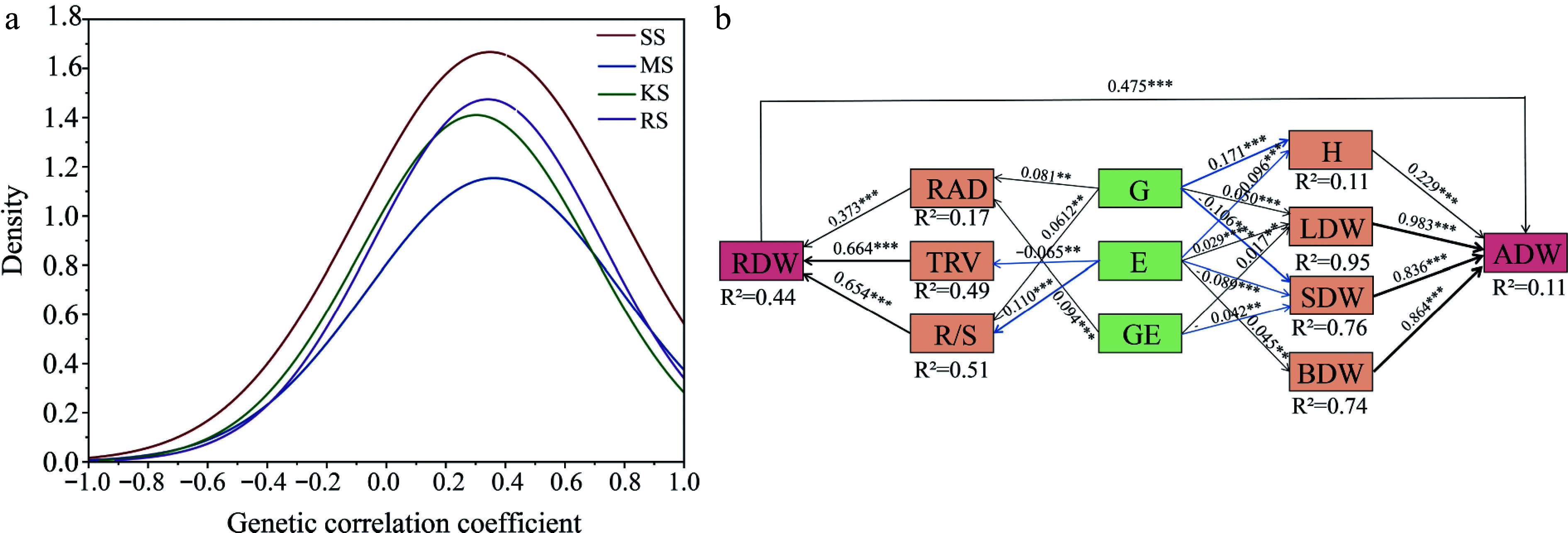
(a) Frequency distribution of genetic correlation indices between all traits. The frequency of distribution of correlation coefficients, measured as genetic correlation coefficients between two indices, was calculated 91 times using 14 traits. (b) Partial least squares structural equation modeling (PLS-SEM) reveals direct and indirect effects of genotype and environment on growth and root function traits. AIC = 36.18, Fisher's C = 41, *p *= 0.072 in the model, Solid blue arrows indicate negative paths (*p* < 0.05), solid black arrows indicate positive paths (*p* < 0.05), and the width of the arrows indicates the strength of the causal relationship. Numbers on the line adjacent to the arrows are standardized path coefficients. *R*^2^ indicates the total variance explained by the model. H, Plant Height; ADW, Aboveground dry weight; LDW, Leaf dry weight; BDW, Branch dry weight; SDW, Stem dry weight; RDW, Root dry weight; RAD, Root average diameter; TRV, Fine root volume; R/S, Root to shoot ratio; * *p* < 0.05; ** *p* < 0.01; *** *p* < 0.001.

The study aimed to investigate the potential genetic covariance among plant traits across different soil environments, as measured by the genetic correlation coefficient (Fig. 7a). The average correlation between traits was found to be positive (average genotype = 0.311). The distribution of correlation coefficients approximated a normal distribution, with many pairwise correlations being statistically significant. This suggests that the growth of various families and the high variability of specific root traits across different soil types may play a crucial role in plant adaptation strategies. Co-variability and plasticity led to the growth traits of Chinese fir at the seedling stage, favoring resource acquisition strategies (Figs 6 & 7a). For instance, in RS soil and SS soil, which possess relatively poor resources, the range of trait variation is greater, and the correlations among different traits are stronger (Table 1).

The results of the structural equation model (SEM) indicate that RAD, TRV, and R/S are key traits influencing the variation in RDW. The genetic effect (G) and the genotype-by-environment interaction (G × E) contribute to RDW through their impact on RAD, while both G and the soil environmental conditions, E, influence RDW through their effect on R/S. Furthermore, the E affects RDW by altering TRV. LDW and SDW are jointly influenced by G, E, and G × E interactions. The G and E effects impact ADW through their influence on H. Overall, RDW does not directly affect plant height (Supplementary Fig. S1), but it can directly influence ADW (Fig. 7b). When utilizing G, E, and G × E effects to assist in selecting for aboveground growth and root functional traits, a clearer understanding of how these factors drive biomass accumulation can be achieved.

## Discussion

### Analysis of variation characteristics and effects

The study comprehensively quantified the responses of multiple aboveground and belowground traits to various soil environments. The results demonstrated that genotype effects, soil environment effects, and G × E interaction effects contributed to variations in the growth and root traits of Chinese fir to differing extents. Several traits exhibited substantial variation across transplantation environments, providing valuable insights into the strategies employed by this species to adapt to diverse soil conditions^[41,42]^. These findings align with previous observations of significant phenotypic variation among different provenances, families, or clones of Chinese fir across forests of varying ages^[3]^. However, unlike earlier studies, this research dissected the contributions of aboveground and belowground organs, enabling a more precise determination of the sources of variation. The analysis revealed that soil conditions and G × E interaction effects are the predominant factors influencing seedling growth, whereas genotype effects were comparatively less significant. This suggests that after three generations of genetic selection, differences in growth among Chinese fir varieties have narrowed. The generalized heritability of tested traits ranged from 0.10 to 0.72, with root functional traits generally showing values below 0.3 (Table 1). This highlights that for multi-generational improved varieties of Chinese fir, maintaining high growth should be coupled with a greater emphasis on leveraging the synergistic benefits of genotype-environment interactions, particularly in optimizing adaptation to varying soil conditions^[43]^.

Analysis indicates that soil environmental variation is the primary factor influencing family ranking and trait plasticity (Fig. 3). This variability suggests that different genotypes of Chinese fir possess distinct genetic potentials for perceiving soil nutrient conditions, regulating root morphology (e.g., fine root ratio, specific root length, and total root volume), and activating soil phosphorus (e.g., through the secretion of acid phosphatase)^[44]^. For instance, phosphorus-efficient clones of Chinese fir respond to low-phosphorus stress by exhibiting root morphological plasticity (such as an increased fine root proportion) and physiological regulation (e.g., secretion of acid phosphatases). Under nitrogen deposition, high nitrogen input alters root exudate composition and enhances phosphorus activation efficiency; however, the specific strategies employed vary among genotypes^[44]^. Similarly, Yang et al. observed that in heterogeneous phosphorus environments, phosphorus-efficient clones of Chinese fir tend to increase root biomass and root length to exploit phosphorus-rich patches, whereas phosphorus-sensitive families rely more on enzymatic phosphorus activation strategies, resulting in limited biomass accumulation^[45]^. This trade-off aligns with the plant economics spectrum theory, which proposes a differentiation between resource-acquisitive strategies (investing in aboveground parts in nutrient-rich environments) and resource-conservative strategies (investing in belowground organs in nutrient-poor environments)^[45,46]^. These findings support the conclusion of this study that, in nutrient-poor soils, plants preferentially increase investment in fine roots within the first three diameter classes to optimize the balance between root functionality and nutrient acquisition.

### Organ allocation and plasticity response

The plant height and aboveground biomass in KS soil are the highest, characterized by strong root branching and larger average root diameters. This suggests that in broadleaf artificial forest soil, increasing root density can expand the area available for nutrient foraging, thereby enhancing the root system's ability to acquire water and nutrients. This increase in root thickness allows for greater nutrient storage, representing a key manifestation of phenotypic plasticity^[47−49]^. Root morphology traits, including root diameter, specific root length, specific root area, and root dry matter content, comprehensively reflect the physiological status of roots and their responses to environmental changes^[50]^. A high specific root length improves the efficiency of resource absorption relative to plant biomass investment, thus boosting the root's nutrient uptake capacity and reflecting the plant's allocation of matter and energy to the underground system. In nutrient-poor soils, plants typically increase their investment in underground dry matter to enhance adaptability to the environment^[51,52]^. This research indicates that root biomass across the four soil types constitutes 17.10% to 24.08% of the total biomass (Fig. 4b). In both SS and RS soils, the anticipated increase in root dry matter content did not materialize; instead, these soils exhibited an increase in SRL and SRA to a certain extent. Interestingly, the total length, surface area, and volume of fine roots in SS and RS soils were not the greatest (Fig. 5d–f). However, when examining the proportion of fine roots across different diameter classes, the proportion of fine roots in the D1–D3 diameter range (0 mm < D ≤ 1.5 mm) was higher, indicating that the overall proportion of fine roots was not the largest (Fig. 5). These findings suggest that changes in root morphology may be more significant than the allocation of root biomass^[26,32]^. Ultimately, these results enhance the understanding of how plants respond to various environments and raise expectations for the plasticity of root functional traits^[22]^.

Recent studies have emphasized the importance of simultaneously considering multiple trait responses to better understand nutrient acquisition strategies in both aboveground and underground plant organs^[12]^. Plants do not respond to environmental changes by altering a single trait; rather, they balance a combination of traits to adapt to these changes. The interplay of multiple traits determines a plant's life history strategy^[23,53]^. Theoretically, there may also be trade-offs between the plasticity responses of various traits, influenced by genetic and ecological (physiological) factors^[54]^. If plant plasticity can be reliably characterized through easily measurable fine root traits, more accurate simulation and prediction of plant behavior may be possible, particularly in response to environmental changes^[18,55−57]^. For instance, RAD is closely linked to SRA, SRL, and RDW, making it a strong predictor of underground foraging strategies. In nutrient-poor soils, root systems tend to be thinner, and plants rely more on alterations in root morphology (such as SRL and SRA) to enhance nutrient acquisition by expanding the soil exploration area—indicating a resource acquisition strategy. However, due to soil heterogeneity and limitations in current technologies, research on root functional traits and their interrelationships lags behind research on aboveground functional traits^[42]^.

### Resource acquisition strategy

As an important timber species, Chinese fir faces challenges in establishing large-scale plantations due to its adaptation to soil environmental changes, including low phosphorus availability, acidification, and continuous cropping obstacles. Similar to other plants, Chinese fir enhances its soil nutrient absorption capacity by modifying root morphological traits. For instance, in this study, seedlings grown in relatively infertile RS and SS soils exhibited increased proportions of fine root length and surface area within the D1, D2, and D3 diameter classes (Fig. 4). This enhancement in fine root proportion and total root surface area facilitates more efficient nutrient uptake. However, variations in fine root biomass may relate to different resource allocation strategies. The SS soil in this study exhibited the lowest fine root biomass but the highest specific root length and specific root area, along with a finer average root diameter. Typically, fine roots with a high specific root length and low root tissue density indicate a rapid resource acquisition strategy. In contrast, evidence suggests that Chinese fir fine roots may adopt a more conservative strategy^[48]^. Adaptability to nutrient-poor soils may also vary in root functional traits following multigenerational selection. Studies indicate that Chinese fir demonstrates high phosphorus uptake and utilization efficiency under low-phosphorus conditions^[57]^. Furthermore, its widespread distribution in acidic soils implies the presence of specialized acid-tolerance mechanisms, such as resistance to aluminum toxicity^[58]^. Root exudates (e.g., organic acids, phosphatases) can influence nutrient solubility and bioavailability. Notably, fluctuations in root exudate flux positively correlate with fine root SRL^[59]^, supporting the 'resource acquisition strategy' hypothesis, which posits that high-SRL roots rely more heavily on exudates for nutrient mobilization.

The strong correlation between traits with different functions reveals the trade-offs and synergistic effects that constrain and coordinate plant functions. In relatively nutrient-poor RS and SS soils, trait variability is greater, and the correlations between different traits are more tightly linked (Fig. 7a). High SRA and SRL are typically negatively correlated with other traits that promote rapid growth, such as branch strength and root diameter. Overall, most root morphological traits exhibit strong correlations, and variation in these traits serves as a key strategy for plants to acquire soil nutrients. This variation drives the differentiation of root architecture and morphological features. Given the multifaceted objectives of forestry production, when considering multi-trait selection and improvement from a breeding perspective, it is essential to consider the complex interactions introduced by G × E when evaluating multi-trait selection and improvement from a breeding perspective. Different traits may exhibit distinct G × E patterns. Consequently, the study of G × E effects in multi-environment, multi-trait forest systems is likely to become a major trend and research hotspot in the future.

## Conclusions

For multi-generational genetically improved varieties, this study explored how genetic effects, soil environmental conditions, and genotype-by-environment interactions contribute to the selection of aboveground growth and root functional traits, thereby enhancing the understanding of their roles in biomass accumulation. The main findings are summarized as follows: (1) soil type and genotype-by-soil interactions predominantly influence the expression of key traits in Chinese fir seedlings, including plant height, biomass, and root functional characteristics, with genotypic effects playing a relatively minor role. Phenotypic traits, especially plant height and biomass, exhibit high plasticity across different soil environments, accompanied by clear rank changes among varieties; (2) different soil types significantly alter aboveground-belowground growth dynamics by regulating root functional traits. In nutrient-poor soils, plants preferentially increase fine root investment within the first three diameter classes (0 mm < D ≤ 1.5 mm) to optimize the balance between root function and nutrient acquisition, resulting in strengthened correlations among root traits and modifying growth patterns during the rapid growth phase; (3) root average diameter (RAD), total root volume (TRV), and root-to-shoot ratio (R/S) were identified as key traits governing variation in root dry weight (RDW). Although RDW does not directly affect plant height, it exerts a direct positive influence on aboveground dry weight (ADW), underscoring the critical role of belowground resource acquisition in driving aerial biomass accumulation.

## SUPPLEMENTARY DATA

Supplementary data to this article can be found online.

## Data Availability

The datasets generated during and/or analyzed during the current study are available from the corresponding author upon reasonable request.
