# Supplementary material for: Growth strategies and phenotypic plasticity of improved Chinese fir families across soil types
Source: For Res (Fayettev). 2025 Oct 30;5:e024. doi: 10.48130/forres-0025-0022 (PMC12647935; doi:10.48130/forres-0025-0022)
Supplement: Supplementary file 1 — Supplementary data to this article can be found online. [file FR-2025-5-0022-Supplementary.zip › 10.48130_forres-0025-0022-Suppl-TableS1.pdf]

Supplementary table 1. Information on the source of family numbers. The breeding procedure was to create seeds by hybridization among parents from different origins, and after the establishment of the assay forest, selecting the best single plants, then collecting spikes, grafting and breeding, and constructing a seed orchard population, which was carried out in three rounds of selection (almost 30 years) according to the breeding procedure, these family numbers are seeds collected by free pollination in the seed orchard.

Family numbers	Production number	Genotype origin (parent information)	One-year old seedling height cm (±SD)
1	yw1	FuJian、ZheJiang	20.56 (±0.82)
2	B10	HuNan、JiangXi	21.42 (±1.21)
3	B111	HuNan、JiangXi	20.18 (±1.15)
4	yw177	FuJian、ZheJiang	22.16 (±1.06)
5	B101	HuNan、JiangXi	20.75 (±0.92)
6	B42	HuNan、JiangXi	20.73 (±0.86)
7	B155	HuNan、JiangXi	21.11 (±1.01)
8	B56	HuNan、JiangXi	20.88(±0.78)
9	K3	ZheJiang	19.83 (±0.94)
10	S19	ZheJiang	20.46 (±1.34)
11	C25	GuangXi、ZheJiang	22.25 (±1.58)
12	B148	HuNan、JiangXi	21.63 (±1.28)
13	L15	ZheJiang	20.62 (±1.08)
14	S12	ZheJiang	21.07 (±1.15)
15	B49	HuNan、JiangXi	20.33 (±1.22)
16	S3	ZheJiang	20.37(±1.49)
17	M33	ZheJiang、GuangXi	22.06 (±1.43)
18	yw155	FuJian、ZheJiang	22.25 (±1.55)
19	S2	ZheJiang	21.26(±1.26)
20	B44	HuNan、JiangXi	21.07 (±1.53)
21	B03	HuNan、JiangXi	21.87 (±1.18)
22	Ywc40	FuJian、ZheJiang	22.23 (±1.49)
23	S17	ZheJiang	21.33 (±1.07)
24	T30	GuangDong、ZheJiang	22.06 (±1.22)
25	CK	seed mixture	21.01 (±1.01)
