# Supplementary material for: Growth strategies and phenotypic plasticity of improved Chinese fir families across soil types
Source: For Res (Fayettev). 2025 Oct 30;5:e024. doi: 10.48130/forres-0025-0022 (PMC12647935; doi:10.48130/forres-0025-0022)
Supplement: Supplementary file 1 — Supplementary data to this article can be found online. [file FR-2025-5-0022-Supplementary.zip › 10.48130_forres-0025-0022-Suppl-FigureS1.pdf]

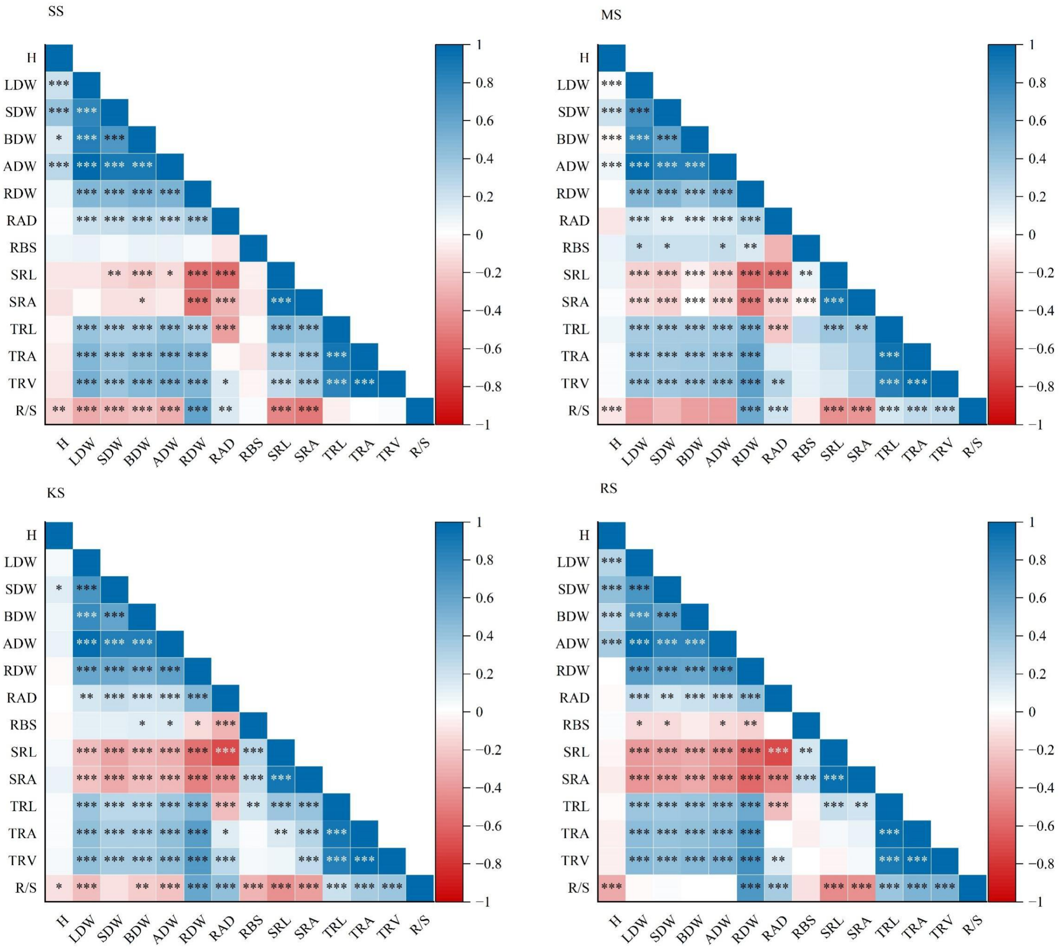

Supplementary figure 1 Heat map showing the correlation between plant morphology and growth indicators. H, Plant Height; ADW, Aboveground dry weight; LDW, Leaf dry weight; BDW, Branch dry weight; SDW, Stem dry weight; RDW, Root dry weight; SRL, Specific root length; SRA, Specific root area; RAD, Root average diameter; RBS, Root branching strength; TRL, Fine root length; TRA, Fine root surface area; TRV, Fine root volume; R/S, Root to shoot ratio.*, $P < 0.05$; **, $P < 0.01$, ***, $P < 0.001$.
